# Impacts of the “transport subsidy initiative on poor TB patients” in Rural China: A Patient-Cohort Based Longitudinal Study in Rural China

**DOI:** 10.1371/journal.pone.0082503

**Published:** 2013-11-25

**Authors:** Qi Zhao, Lixia Wang, Tao Tao, Biao Xu

**Affiliations:** 1 School of Public Health, Fudan University, Shanghai, China; 2 National Centre for Tuberculosis Prevention and Control, Beijing, China; San Francisco General Hospital, University of California San Francisco, United States of America

## Abstract

**Objective:**

To describe the financial burden on TB patients for transportation during treatment, and to evaluate the impacts of the “transportation subsidy initiative on poor TB patients” in rural China for improving poor patients’ access to TB treatment.

**Methods:**

A Case-cohort of 429 TB patients was investigated through questionnaire interviews in four counties of two provinces in China. Information on the financial burden for transportation during TB diagnosis and treatment was collected. Qualitative in-depth interviews with 26 TB patients were carried out to understand their perceptions of transportation subsidy initiative.

**Results:**

The mean transportation cost of TB medical care was 97 CNY (70 CNY in median), varying from 0 to 700 CNY. About 51% of the patients spent more than 10 CNY per round trip to the TB dispensary. Of the 429 TB patients investigated, 139 had received transportation subsidies after getting TB diagnosis; 15/139 (10.9%) showed dissatisfaction, mainly because the subsidy amount being insufficient. The income of patients receiving transportation subsidies was significantly lower than those not receiving the subsidies (*p*<0.05). The impression that an appropriate transportation subsidy enables patients to complete the required visits during their TB treatment was obtained after observation of over 80% of the patients.

**Conclusion:**

The transportation subsidy plays an important role in reducing financial burden on poor TB patients for the completion of treatment. However, the coverage was limited and the amount of subsidy was not enough under the present policy. Considering the poverty of rural TB patients, a universal coverage and a rational amount of transportation subsidy should be proposed.

## Introduction

Tuberculosis (TB), the world’s leading cause of death due to a single infectious agent among adults [1], is a disease associated with poverty with a disproportionate burden of disease falling heavily on low and middle income countries. China has the world's second largest tuberculosis case burden with more than 1.3 million new cases every year, mainly distributed in poor rural areas [2]. The mortality rate from TB in poor rural areas is nearly three times higher than that in economically developed urban areas [3]. This difference is mainly due to factors associated with poverty and under-development, such as poor living conditions, underlying low health and nutritional status, lack of money to pay for health care and inadequate access to health services [4]. Financial barriers to accessing TB treatment have been identified as the key factor in non-compliance and treatment default in rural China [5], thus contributing to an increased prevalence of drug resistant tuberculosis.

The China National TB control program provides TB diagnostis and treatment through sputum smear microscopy, X-ray examination, and 6-8 months of anti-TB treatment with a standardised regimen free of charge. However, the basic unit of TB medical care is county TB dispensary (CTD) which is usually far away from a patient’s home in rural areas. Most of the counties have only one CTD serving a population varying in size from less than one hundred thousand to more than one million [6]. Patients pay a substantial proportion of their income for accessing the free anti-TB services; this includes costs for transportation to and from the CTD, and payments of other medical costs [7].During the treatment, some patients, particularly those in the poor and vulnerable groups, may drop out completely at any stage. Studies of access to TB care in Viet Nam, Malawi and other low-income countries have reported that charges for consultation, diagnosis tests and treatment medicines are not affordable in many cases [8–10]. After getting the TB diagnosis, the expenditures on transportation, accommodation and subsistence could also become a heavy burden on patients [11,12].

The Stop-TB Partnership has set a global target of curing 85% of all diagnosed smear-positive TB patients by 2015 [13]. In order to effectively control TB epidemic and rapidly achieving the global target, many countries have been searching for strategies that are adapted to local TB epidemiology and control policies [14]. As a result, interventions aiming at improving the quality of primary care services and increasing community-based care, involving private providers, strengthening the responsibility of public health institutions and making special efforts to reach vulnerable populations, have been proposed and implemented in different settings [15]. Among these efforts, interventions using enablers and incentives have been considered effective in improving TB care. Enablers remove barriers to seeking quality-assured health care services through provision of financial or material help to patients, while incentives provide further stimuli to beneficiaries to achieve a desired outcome [16].

In December 2007, a pro-poor “transportation subsidy initiative (TSI) to poor TB patients” was proposed to be carried out in 16 relatively poor areas of China, where the TB control program was supported by a World Bank loan together with the UK Department for International Development (the World Bank/DFID China TB Control Program). The TSI aimed to support poor TB patients in completing treatment course through providing a transportation subsidy as an enabler. The targeted populations were TB patients facing economic difficulties in transportation for treatment visits. The coverage of TSI was estimated as 30% of all TB patients in each county, which could be adjusted in each province based on the total amount of funds available and the local economic conditions. Under the TSI project, poor TB patients received 10CNY per visit as a transportation subsidy for the monthly visit to the CTD. In total, a subsidized patient could receive 60CNY (for newly diagnosed patients) and 80CNY (for retreated TB patients) respectively during the 6-8 months treatment course [17]. At the end of 2008, TSI was carried out in 12 provinces covered by the World Bank/DFID China TB Control Program. 

This study aimed at describing financial burden on TB patients for transportation to and from the CTD during treatment, and evaluating the impacts of the “transportation subsidy initiative to poor TB patients” in rural China on improving poor patients’ access to TB treatment. 

## Methodology

### Study design

The study was carried out in Chongqing City and Fujian Province. These two areas were in the first group for which the TSI was launched in August 2008. In each place, 2 counties/districts were selected as study sites in consideration of representativeness in demographics, socio-economic status and cultural characteristics, such as income per capita, population size, proportion of agricultural population and people living under the national poverty line, TB report rate and cure rate in 2006, as well as the willingness to participate in the study. Finally, Xianyou and Datian County in Fujian, and Wanzhou and Shapingba District in Chongqing were selected as study sites. The report rate of newly diagnosed TB of these four counties were 64.97/10^5^, 58.36/10^5^, 82.50/10^5^, and 94.02/10^5^ respectively.

Both quantitative investigation and qualitative interview were applied in the 4 sampled counties/districts from April 2009 to February 2010. The subjects of the study were all active pulmonary TB patients diagnosed in the CTD and during the course of treatment at the time of investigation.

All active TB patients diagnosed within 5 months prior to the initiation of the study in the sampled 4 counties were study subjects. They were enrolled and interviewed face to face by the TB management staff in the clinics. Enrolling of participants was stopped while reaching research sampling size. Follow-up interviews were given retrospectively and prospectively in the 1^st^, 2^nd^, 5^th^ and 6^th^ or 8^th^ month of the treatment course.

Participants of qualitative study were recruited using purposive sampling technique to cover patients receiving and not receiving transportation subsidy, female and male. Finally, a total of 26 eligible individuals were interviewed; of them, 14 received and 12 didn’t receive transportation subsidy. The ratio of male to female was 17 to 9.

### Sample size

In this study, catastrophic health expenditure was defined if patient’s expenditure for TB care is equal to or greater than 30% of their average annual expenditure. Assuming that at least 50% of TB patients incurred catastrophic health expenditure on TB care, the estimated sample size was calculated as:

$$N={\text{t}}_{\alpha }{}^{\text{2}}\times \frac{p(1-p)}{{\text{d}}^{2}}=1.96^{2}\times \frac{0.5\times (1-0.5)}{0.05^{2}}=384$$

p: proportion of patients who incurred catastrophic health expenditures to the total number of patients. In this study P is estimated as 50%.

α=0.05

d: the allowed bias, 0.1p=0.1*0.5=0.05

### Data collection

The eligible patients were interviewed by physicians in CTDs using a structured questionnaire. The questionnaire used in this study covered information on patient’s demographics, socioeconomic status (household income, individual income, self-rating of economic status, education, occupation, and health insurance, etc.), and disease profile. Transportation costs associated with TB health care, amount of transportation subsidy received, and patients’ expectations for the transportation subsidy were also collected. Because patients were recruited for the study at various stages of their treatment, information before recruitment was collected retrospectively, while information after recruitment was collected prospectively during patients’ monthly visit to the CTD until treatment was completed. The questionnaire has been used in several previous studies with access to TB care in rural China, and modified for this specific project accordingly [18]. 

A theme guide was developed for in-depth individual interview. The main topics were patients’ ability to pay or co-pay for transportation, perceptions in the impact and benefit of TSI, experiences on and difficulties in receiving transportation subsidy, and awareness, satisfaction and expectations of TSI.

**Figure 1:**
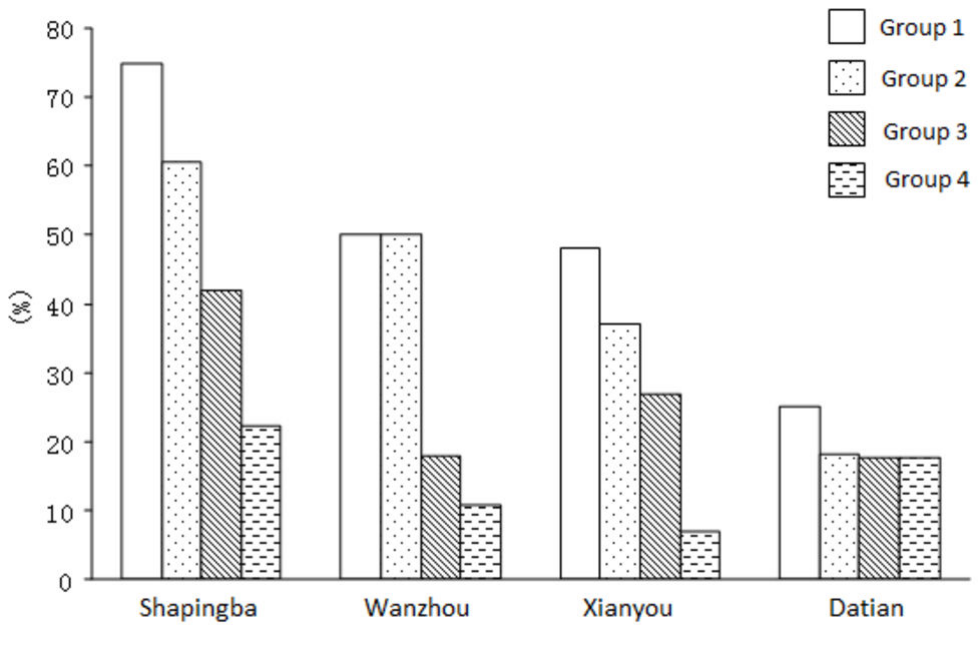
Proportion of subsidized patients in different income groups Quartiles (P25~P75) of household income per capita were applied from the poorest 25 percent (Group 1) to the richest 25 percent (Group 4).

### Data analysis

A database established using EPI DATA 3.0.1. SPSS 11.05 (SPSS Inc., Chicago, IL, USA) was used for data analysis. Mean, median, proportion and 95%CI were used in description. Chi-square tests, Kruskal-Wallis test,Mann-Whitney U test and stratification were applied in comparing sub-groups. When measuring the income level of patients, quartiles (P25~P75) of household income per capita, i.e. the household average, were applied from the poorest 25 percent (Group 1) to the richest 25 percent (Group 4). **Transportation cost means** expenditures on transportation to and from CTD for TB diagnosis and 6-8 months’ treatment course including the costs incurred by both the patient and the accompanying persons.

Content analysis with an inductive approach was used for the data from in-depth interview. The transcribed materials were coded line-by-line or paragraph-by-paragraph by research team using MAXQDA2 (VERBI Software, Consult, and Research GmbH, Germany). The codes were clustered together, and similar codes were grouped to create tentative categories and sub-categories, and the main themes emerged based on the patterns and relationship between the categories. 

### Quality control

Each in-depth interview had a moderator and a note-taker who understood the local language. The in-depth interviews were also tape-recorded. The tapes and notes of the in-depth interviews were transcribed into mandarin Chinese within 48 hours by the research team members including the author of this thesis. Later, the transcribed materials were re-checked by postgraduate students in the same department. 

**Figure 2:**
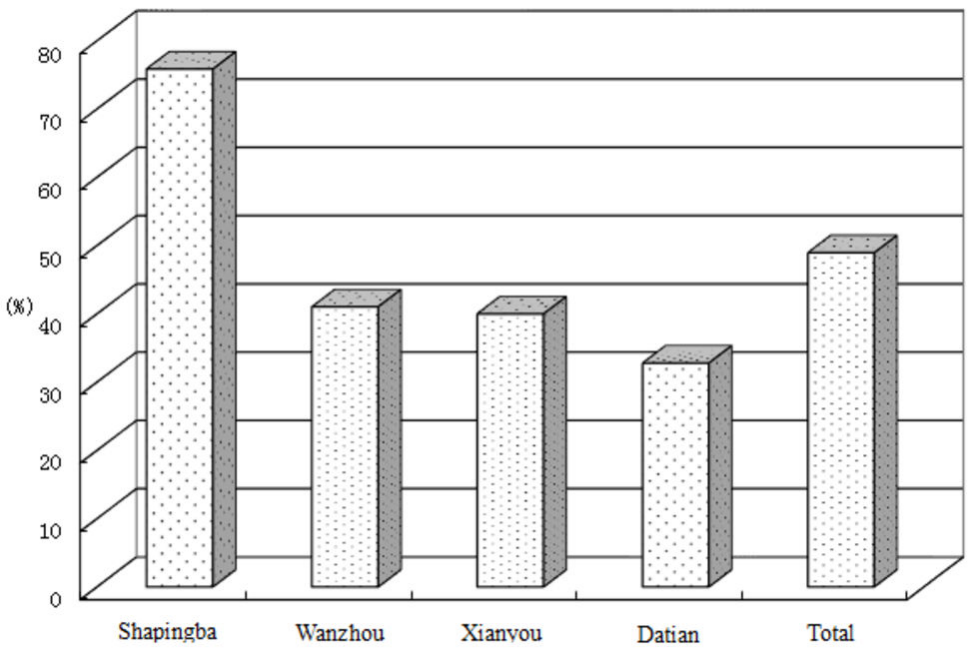
Proportion of TB patients spending less than 10CNY on transportation

All the data collectors and the local project administrators in each county were trained by research team and the objectives, methods, importance of the study and contents of the questionnaires, skill of interviewing were explained, taught, practiced and discussed. The research team revisited the study fields for an average of three days per month during the whole study period to check the complement of questionnaires.

### Ethical considerations

Written consent was obtained from all the participants after a description of the study was given prior to the interview. If the subject was younger than 18 years old, his/her parent or guardian was present on behalf of him/her during the entire reading and discussion of consent andthe parent or guardian signed the consent. If the subject was illiterate, a person who spoke and understood well, but could not read and write, an impartial witness was present during the entire reading and discussion of consent. Afterwards, the subject signed and dated the informed consent. The impartial witness also signed and dated the informed consent along with the individual who read and discussed the informed consent (investigator or study staff personnel).The investigators received training in human subject protection and interview skills. Approval of this study was obtained from the Ethics Committee of the School of Public Health, Fudan University

## Results

### 1: Demographic and socioeconomic data

In total, 429 TB patients under treatment were recruited, of whom 284 were given a diagnosis of smear positive TB; 139 (32.4%) received transportation subsidy. The average age of patients was 43 years, varying from 18 to 85, and 70.2% were male. Forty-five percent of them had attended primary or secondary school, and 84.1% had medical insurance, mainly covered by the recently re-established new rural cooperative medical scheme (NCMS) which in most cases did not cover outpatient expenditures. The mean annual household income per capita was 6,484CNY, with a median of 5,000CNY. The income of patients varied geographically, the lowest of which was in Datian, Fujian Province (4,617CNY) and the highest in Shapingba, Chongqing (9,325 CNY). For this study, the income of subjects in each sampled site was grouped into quartiles from low to high according to the annual household income per capita (Table 1). 

**Table 1:** General information of subjects in the four study sites (CNY).

Items	No.	%
Sites		
Wanzhou	117	27.3
Shapingba	116	27.0
Xianyou	135	31.5
Datian	61	14.2
Gender		
Male	301	70.2
Female	127	29.6
Education		
Illiterate	66	15.4
Primary school (<6yrs)	115	26.8
Secondary school(6-9yrs)	112	26.1
High school(9-12yrs)	83	19.3
College and above	51	11.9
Medical insurance		
Yes	361	84.1
No	68	15.9
Occupation		
Farmer	148	34.5
Student	53	12.4
Non-farmer	228	53.1
TB diagnosis		
Smear +	284	66.2
Smear -	145	33.8
Treatment		
completed	373	86.9
Under treatment	56	13.1
Income group		
1 (<P25)	1631.04	1538
2 (P25~)	3920.54	3750
3 (P50~)	6628.24	6000
4 (P75~)	14311.88	12500
Total	6707.48	5000

Of the 26 TB patients, aged from 16 to 71 years, participating in the in-depth individual interviews, 14 were eligible for transportation subsidies. The ratio of male to female of the participants was 17 to 9. The occupations of the participants were diverse, including: farmers, migrant workers laid-off workers, students, self-employed businessmen, retired and unemployed people. The monthly income of participants varied from zero to thousands of Chinese Yuan (CNY) per month. The interviews were carried out in separate quiet rooms, lasting about 40 minutes. 

### 2: Transportation cost

The transportation cost per round trip to the CTD was, on average, 15.92CNY (median 12CNY). There were significant differences in expenditures on transportation between study sites (Table 2). The lowest cost of transportation was in Shapingba, about 7.36 CNY on average (median 3.5CNY). In the other three sites, the average cost per round trip was about 20CNY (median 16CNY). The mean of patient’s total transportation cost was 97CNY, (median 70CNY), varying from 0 to 700CNY. Besides different distance, the variation of transportation costs between study sites was also due to differences in frequency of required follow-up visits. Patients from Wanzhou County only visited TB dispensary 3~4 times during treatment, whereas in the other three counties/districts, patients were required to visit the TB dispensary every month, which meant at least 6 times for newly diagnosed patients and 8 times for retreated ones. Thus, although the transportation cost per round trip in Wanzhou was higher than in the other study sites, the total expendituresthere was significantly lower than in Xianyou and Datian (*p*<0.01).

### 3: Transportation subsidy

Sixty percent of the investigated patients thought that the transportation subsidy should be provided to elderly people without children, the "five-guarantee family" (five guarantees families means childless and infirm old persons who are guaranteed by the government for food,  clothing,  medical care,  housing and burial expenses. They are poor residents supported by social insurance in rural China), the certified low-income families and the unemployed, whereas 48.2% of the subjects considered the subsidy should cover the whole TB patients. After grouping patients’ household income per capita, it was found that patients in the lower-income group were more likely to receive transportation subsidy in all four study sites（Figure 1). The household income per capita was 4,660CNY on average for subsidized patients and 7,790CNY for those not subsidized, with the median at 4,000CNY and 6,000CNY respectively (Table 3). The income of patients receiving the subsidy was significantly lower than those without subsidies (p<0.05).

**Table 2:** Transportation costs of TB patients in each study site (CNY).

Site	Visit times	Cost per round trip						Total transportation cost						
		Mean	Median	P25~P75		*p* ^*a*^		Mean	Median	P25~P75		Min	Max	*p^a^*
Shapingba	6-8	7.36	3.5	2	10	<0.001		55	27.5	14	80	0	350	<0.001
Wanzhou	3-8	19.18	16	5	30			79	64	20	120	0	400	
Xianyou	6-8	17.19	15	10	20			121	105	70	140	0	490	
Datian	6-8	23.08	20	6.5	40			162	140	46	280	0	700	
Total		15.92	12	4	20			97	70	28	140	0	700	

a: *p*-value from Kruskal Wallis Test

According to the implementation guideline “Transportation Subsidy Initiative to poor TB patients”, the subsidy for transportation per round trip was 10 CNY. Among the patients who had received subsidies, 51% of them spent more than 10CNY per round trip. The proportion was as low as 16.9 % in Shapingba, and about 70 % in the other 3 study sites (Figure 2). The majority of participants of the study, regardless of whether receiving or not receiving the transportation subsidy, perceived that an appropriate transportation subsidy could enable patients’ to visit CTD during treatment as required and improve the treatment completion. Only 15 (10.9%) patients showed dissatisfaction with the TSI, mainly because the subsidy amount was regarded as insufficient. Eighteen (20%) patients of those who received subsidy thought that the subsidy was not useful in enabling TB patients’ healthcare seeking and/or enabling TB patients’ adherence to treatment Of them 17 (95%) were from Shapingba District. Similar results had been found in the qualitative interviews. 

Findings from the in-depth interviews showed that TSI was warmly welcomed by patients. All patients praised the advantages of TSI, considering it as humanistic and having shown the concern of the government to poor patients. Although the amount of subsidy was small, it was very useful to really poor patients. Without the subsidies, poor TB patients would have to seek financial helps or assistances to continue the treatment, such as borrowing money for taking a bus, walking or biking for several hours to the CTD, combining several means of transport for low cost, or seeking financial support from the local village, town or government. Expect one patient who considered transportation subsidy as useless, all the interviewees praised the positive effects of TSI on poor TB patients.

“The transportation subsidy was helpful to poor people like us! Rich people didn’t care about 60 Yuan, but it meant a lot to me.” (Female TB patient)

“I was happy after receiving transportation subsidy; otherwise I had to pay all transportation fees by myself……sometimes I failed to visit the TB dispensary for lacking money.” (Male TB patient)

According to the participants, the effects of the transportation subsidy depended on both the extent of poverty and the distance between patients’ home and CTD. Most of the participants reported that for patients living nearby, transportation subsidies could cover all the expenditures on transportation, while for those who lived in remote mountainous areas, 10 CNY per month could hardly cover the expenditures, which could be tens to hundreds of Chinese Yuan. Hence the effect of transportation subsidies was much more apparent for patients living nearby. The effects of TSI were perceived by the participants as 1) the subsidy had no effect on non-impoverished patients; 2) the subsidy had certain positive effects on common poor patients (the majorities in our study), but it had limited influence on their health seeking behaviors; 3) the subsidy had significant positive effects on very poor patients, because sometimes they were unable to visit CTD for lack of transportation fees, especially the few extremely poor patients. Consequently, the transportation subsidy could play its role in enabling patients to complete TB treatments on the basis of the identification of the really poor population.

“Even one Yuan of subsidy was useful to rural residents, especially to those old, who were unable to work and getting no money from their children. I had income and my children earned money as well, so the transportation subsidy was less important for people like me.” (Male patient)

“I had to pay 30 Yuan for a round trip. It was a bit expensive, but what can I do? I had to come here no matter how expensive it was.” (Male patient)

“We got up early in the morning and went to the TB dispensary on foot for saving money. If the sunshine was too strong, we would go by minibus which cost us 1 Yuan. We would never choose those bus charging 1 Yuan and 50 Cent.” (Female patient)

**Table 3:** Annual incomes per capita of TB patients receiving or not receiving transportation subsidy (CNY).

	Patients receiving subsidy			Patients not receiving subsidy					
							Total		
Site	subsidy			subsidy					
	N	Mean	Median	N	Mean	Median	N	Mean	Median
Shapingba^a^	57	6073.7	5333	57	12575.4	10000	114	9324.5	7200
Wanzhou^a^	36	2912.5	2367	76	5424.7	5000	112	4617.2	3917
Xianyou^a^	32	3512.4	3000	77	7079.2	6000	109	6032.0	4500
Datian	12	6251.4	5003	49	7010.9	5000	61	6861.5	5000
Total^a^	137	4660.3	4000	259	7790.4	6000	396	6707.5	5000

a: Kruskal Wallis Test p<0.05

## Discussion

Under China’s national TB control program, patients should visit the TB dispensary every month or at least 4 times during the 1^st^, 2^nd^and 5^th^month of treatment course. The transportation costs for treatment visits varied geographically, and it was reported that for the poor TB patients, lack of money for transportation might result in treatment defaults [6]. It was found in this study that the average transportation cost for TB patients was 97 CNY. About 51% of the patients spent more than 10 CNY per visit to the TB dispensary. 

Transportation cost was a real cost, sometimes the only cost which could not be avoided by TB patients. As mentioned above, patients should and must visit the CTD monthly or at least three times during their treatment course to receive medicines and smear tests [19]. In some poverty-stricken areas, especially remote and mountainous regions, poor patients live far away from the CTD, and the necessary transportation expenses became unaffordable. Some TB patients cannot adhere to their regulated follow-up visits, or even gave up treatment due to lack of funds for transportation [20,21]. In Hubei province of China, Chen [20] reported that the rate of cases with limited access (LA) to county TB dispensary (CTD) among new smear positive (NSP) is higher in the group living beyond 30 km away than in the group living 25-30 km (χ^2^ =7.063, P<0.05 ) away, the rate of case with LA among the NSP cases is higher in mountain area than in hill area (χ^2^ =6.085. P<0.05 ) and in plain area (χ^2^ =4.5, P<0.05 ). And Chen pointed out that the big service scope of TB dispensary and bad traffic conditions in the remote and mountain area force patients to spend much time and cost on transportation, resulting in poor adherence of patients to TB treatment and failure of a big proportion of patients who are likely to seek health service in village clinics if transportation is not a problem. 

Performance-based incentives for both patients and providers have been integrated into many TB control programs, with the aim of increasing case detection and improving treatment outcome, interrupting transmission of TB and improving the quality of TB services [22]. In recent years, such incentive measures have been adopted by an increasing number of countries. According to a worldwide survey by Mookherji, there were 33 types of incentive (or enabling) measures incorporated in TB control projects in 48 regions or counties during 2003-2005; of these, 16 were for patients and 17 for health providers [15,23].

Following direct cash transfers for by patients to increase TB treatment compliance, transportation subsidies were the second most frequently used enabler for TB patients. Providing a transportation subsidy could potentially encourage TB suspects to seek health care early, attend periodic follow-up visits and get medications on time resulting in higher treatment compliance and cure rates. Davidson and colleagues carried out an intervention study in the USA, and found that giving an additional transportation subsidy to TB patients increased their treatment compliance [24]. In the evaluation of a FIDELIS project that provided transportation subsidy to TB patients in Shanxi Province China,Hu and colleagues found that the number of new smear positive TB cases detected had increased significantly (from 5,105 to 7,737) while cure rate remained high at 93% [25]. Under China’s strengthened DOTS program (since 2002), treatment completion has reached 90% and above, although there are still difficulties for patients to complete their treatment.Poor TB patients had to seek financial help or assistance to continue treatment, such as borrowing money for taking a bus, combining several transportation means or even walking for low cost, or getting financial support from the government. In our study, although all patients completed their treatment, more than 80% considered that an appropriate transportation subsidy was helpful in enabling patients to complete the required treatment visits, especially for the poorest group and the patients living in remote and mountainous areas. 

According to current epidemiological trends, non-targeted interventions for case detection and treatment adherence will not contribute to achieving the tuberculosis control targets and reducing poverty [26]. Only by taking the needs of the poor and vulnerable population into consideration (i.e. the high risk population for TB), by improving their access to health care and equity, and giving more and specific support, could more TB patients be detected and cured effectively [27].

An important finding in this study is that patients receiving subsidies had a significantly lower income (4,000 vs. 6,000 CNY) compared to those not receiving the subsidies. This reflected the orientation of the Transportation Subsidy Initiative was to help especially poor TB patients. Yet, not all patients, not even poor patients, could receive subsidy,due to limited money and limited coverage. In our study, about half of the patients in the lowest income group did not receive a transportation subsidy.More than 50% of our patients had an annual income per capita lower than the national average (4,096CNY). According to the report of the fifth national epidemiology survey of Tuberculosis, 82% of TB patients’ income were lower than the local average income , especially of patients in rural area[28].

The amount of subsidy is another important issue to consider. The proposed 10CNY per trip (TSI implementation guideline) was not sufficient for the majority of patients. In our study, 51% of the subjects spent more than 10CNY per trip. The current amount only met the transportation needs of TB patients living in urban areas or close to the TB dispensaries, but was not enough to make up for patients living in remote and mountainous areas. Paradoxically, with easy transportation, short distance and relatively high income, the 10CNY subsidy per trip for those patients living in urban cities might weigh less than for patients from the rural and mountainous areas.

## Limitations

The TSI project has been carried out only in part of the provinces covered by the “World Bank/DFID China TB Control Program” project, with big diversities in the cover range and extent of implementation. Therefore, the 4 counties with relatively long duration of implementation and broad coverage of TSI were selected purposefully as study sites. However, compared to the total of more than 2600 counties in our country, the sample size is small, limiting the generalization and application of study results.

Constrained by time, a longitudinal study using a case-cohort study was introduced in analyzing the economic burden of TB patients. During the investigation, patients had to recall previous healthcare seeking experiences and the expenses of each visit. So the recall bias is inevitable, especially for those patients recruited in the late stage of their treatment periods. To avoid such bias, patients were required to present their invoices or related vouchers, while health facilities were asked to provide all kinds of examination sheets. Nevertheless, transportation costs were relatively fixed, therefore incurred less recall bias. Before the end of data collection, the extreme values were checked and the corresponding patients were re-visited. 

## Conclusion

For poor TB patients, the transportation subsidy plays an important role in enabling their treatment completion. However, the enabling effects of the Transportation Subsidy Initiative were constrained by the low amount of subsidy and the limited coverage. For poor rural TB patients, a universal coverage and a workable amount of transportation subsidy should be taken into consideration.

## Supporting Information

Appendix S1 **Questionnaire.**
(DOC)
