# Supplementary material for: Impacts of the “transport subsidy initiative on poor TB patients” in Rural China: A Patient-Cohort Based Longitudinal Study in Rural China
Source: PLoS One. 2013 Nov 25;8(11):e82503. doi: 10.1371/journal.pone.0082503 (PMC3839919; doi:10.1371/journal.pone.0082503)
Supplement: Appendix S1 — Questionnaire. (DOC) [file pone.0082503.s001.doc]

**Annex1: Questionnaire**

Questionnaire on tuberculosis diagnosis and treatment history

code：□□□□□□□

Dispensary：___________；Investigator：____________；Date：_____/___/___

**Part Ⅰ. General information**

1． Gender: ⑴male ⑵female;

1.1 Date of birth date (yyyy/mm/dd) _____/___/___;

1.2 Residence: county ______town ______village ______； 1.3 Tel: ____________

1.3 In which province / municipality / autonomous region were you Hukou? _________

2．Education: ⑴never been to school ⑵below primary school ⑶primary school

⑷junior high school ⑸ senior high school or occupational education

⑹college and above

3．Marriage：⑴unmarried ⑵married ⑶ widow/widower ⑷divorced

4．Occupation：⑴ farmer ⑵ worker in a factory in a village or town ⑶ cadre, teacher or health worker of a village ⑷ student ⑸ family business ⑹ labor workers outside the town ⑺ government employee ⑻ others:_______

5．What kind of medical insurance do you have:

⑴ city/town social insurance ⑵ New rural Cooperative insurance ⑶ commercial medical insurance ⑷ no any medical insurances ⑸ others:_______

6．In the past year, have you had a job outside your town?

⑴ Yes ⑵ No (skip question #7)

7．What kind of your current job is? _______________.

How long did you have this job? From _____/___/___ to _____/___/___，

8 .BCG vaccination history:_________

**Part Ⅱ. Social economic information**

9. Family size: ____ (persons);

10.1. Number of family members able to work: ____(persons)

10.2 The primary source of household income comes from:

⑴ the patient ⑵ other persons ⑶ both the patient and other persons

10. The patient is: ⑴ able to work ⑵ a supported member

11. The farmland owned by your family: ____ (acres);

11.1 Major products: ⑴ grains ⑵ vegetables ⑶ fruits ⑷ others:_______

11.2 Number of breeding poultry: ___;

11.3 Number of domestic animals: pigs/sheep___, cattle___.

12. Family income per year _______ (CNY);

12.1 Does your family obtain government financial aids during recent 3 years? ⑴ Yes ⑵ No

13. Income of the patient per year: _______ (CNY);

13.1 proportion of the family income that comes from the patient:

⑴ <=25% ⑵26%~50% ⑶51%~75% ⑷ >75%

14. The source of income of the patient: ⑴ farming ⑵ full time non-farming work in town ⑶alternative family business ⑷individual business ⑸ supported by children ⑹ national alms ⑺ hard physical work outside the town ⑻others:_______

15. Which part of the patient’s income will be lost due to having tuberculosis?

⑴full time work in town ⑵ farming ⑶ alternative family business ⑷individual business

⑸ work outside the town ⑹ others: _______

**Part Ⅲ. Information on current treatment**

16. The result of 1^st^ 3 sputum smear tests: evening____; early morning_____; on spot______

17. Date of TB diagnosis / / .

18. Types of TB diagnosis:

(1) Pulmonary TB, SS+, without previous TB treatment history

(2) Pulmonary TB, SS -, without previous TB treatment history

(3) Pulmonary TB, SS+, having previous TB treatment history

(4) Pulmonary TB, SS -, having previous TB treatment history

19. Stage of TB: (1)Ⅰ (2) Ⅱ(3) Ⅲ (4) Ⅳ

20. Results of Chest X-Ray examination:

21. Cavities: (1) Yes (2) No

22. Hemoptysis: (1) Yes (2) No

23. Main symptoms of current episode: (1) cough (2) expectoration (3) hemoptysis (4) fever

(5) chest pain (6) fatigue (7) night sweating (8) no symptoms (9) others

24. How long has the cough (if having) lasted?

1) <=1wk (2) 2wk~ (3) 3wk~ (4) 1 month~ (5) 2m~ (6) 6m~ (7) >=1yr

25. The reason of visiting TB dispensary: (1) referred by doctors in general hospital/village doctor (2) self referred (3) suggested by other non-health providers

26. If the symptom has lasted for more than 3 weeks, why do the patient not visit TB dispensary?

(1) don’t know TB dispensary while seeking health care in other health facility

(2) know TB dispensary but don’t know having TB while seeking care in other health facility

(3) haven’t sought health care because:

(a)patient did not care (b) no free time, busy working

(c) long distance to TB dispensary (d) lack of money

27. Have you used any anti-TB medicine before? (1) Yes, (2) No (skip to question #29)

28. If you have used anti-TB medicine, when yr month for how long? (month)

29. Have you contacted anyone with tuberculosis during recent year?

⑴Yes ⑵ No (skip to question #31)

30. Where did you contact the person with tuberculosis?

⑴at working place in the town (a colleague having TB) ⑵ at working place while working away from the town ⑶ at home (a family member having tuberculosis) ⑷ at community (a neighbor having tuberculosis) ⑸ others:______

31. Besides tuberculosis, do you have any other chronic diseases (diagnosed lasting over 1 year)?

⑴Yes ⑵ No (skip to question #34)

32. The most serious diseases influencing your living are:

⑴__________ ⑵__________ ⑶__________

33. Present treatment scheme is:

⑴2HRZE/4H_3_R_3_ ⑵2HRZES/4H_3_R_3_E_3_ ⑶2H_3_R_3_Z_3_S_3_E_3_/6H_3_R_3_E_3_ ⑷others:_______

34. Date of treatment initiation: ___/___ /_____.

Part IV. Information on previous TB medical care

| .Items | TB diagnosis/treatment | | | | |
| --- | --- | --- | --- | --- | --- |
|  | 1st | 2nd | 3rd | 4th | 5th |
|  | Present→ recent→ most previous | | | | |
| 35. Date of TB diagnosis (yyyy/mm/dd) |  |  |  |  |  |
| 36. Place of TB diagnosis ⑴county and upper hospital ⑵county TB dispensary ⑶town hospital |  |  |  |  |  |
| 37. Type of diagnosis: (1)SS+ PTB; (2)SS- PTB; (3)Suspected TB |  |  |  |  |  |
| 38. Examination: ⑴fluoroscopy ⑵chest X-ray ⑶blood RTO ⑷sputum smear test ⑸CT  ⑹no examination ⑺other: |  |  |  |  |  |
| 39. Date of treatment initiation (yyyy/mm/dd) |  |  |  |  |  |
| 40. Treatment therapy:  individual therapy using (1) INH (2) RIF (3) streptocomycin (4) ENZ (5) PZA |  |  |  |  |  |
| 41. Treatment therapy using combined medicine  (1) 2HRZE/4H_3_R_3_ (2)2HRZES/4H_3_R_3_E_3_ (3)2H_3_R_3_Z_3_S_3_E_3_/6H_3_R_3_E_3_ (4)other: |  |  |  |  |  |
| 42. Duration of Treatment (month) |  |  |  |  |  |
| 43. Outcome of treatment:  (1) cured (2) failed (3) default |  |  |  |  |  |
| 44. Reasons for treatment default  (1) adverse effect of medicine  (2) left town  (3) lack of money (4) patient don’t care  (5) feel good, no need for persist in treatment  (6) doctor said it was finished |  |  |  |  |  |
| 45. Did you used below medicine during treatment?  1. Fluoroquinolone drugs  2. Kanamycin  3. Capreomycin  4. Amikacin |  |  |  |  |  |
| 46.Expense of examination |  |  |  |  |  |
| 47.Expense of treatment |  |  |  |  |  |
| 48.Expense of transportation and accommodation |  |  |  |  |  |
| 49. Expense of accommodation |  |  |  |  |  |
| 50.Other expenses: |  |  |  |  |  |
| 51. Medical expense covered by collective medical insurance |  |  |  |  |  |

**Part V. Information on health-care seeking before TB diagnosis**

| Items | Health-care seeking | | | | |
| --- | --- | --- | --- | --- | --- |
|  | 1st | 2nd | 3rd | 4th | 5th |
|  | Present→ recent→previous | | | | |
| 52. Main symptom:  ⑴cough ⑵expectoration ⑶hemoptysis ⑷ fever  ⑸chest pain ⑹fatigue ⑺night sweat ⑻routine physical examination ⑼other symptoms:_______ |  |  |  |  |  |
| 53. Date of 1^st^ main symptoms occurred (yyyy/mm) |  |  |  |  |  |
| 54. Date of health-care seeking(yyyy/mm/dd) |  |  |  |  |  |
| 55.Health facility visited:  ⑴county and upper hospital ⑵county TB dispensary ⑶town hospital ⑷village health station ⑸pharmacy and self -medication ⑹ other: |  |  |  |  |  |
| 56. Reason for facility selecting:  ⑴near to home ⑵familiar with the provider  ⑶serious symptoms ⑷light symptoms ⑸high quality of medical treatment ⑹cheap ⑺ other: |  |  |  |  |  |
| 57. Examination: ⑴fluoroscopy ⑵chest X-ray ⑶blood RTO ⑷sputum smear test ⑸CT  ⑹no examination ⑺other: |  |  |  |  |  |
| 58. Diagnosis |  |  |  |  |  |
| 59. Date of diagnosis (yyyy/mm/dd) |  |  |  |  |  |
| 60. Main treatment regimen |  |  |  |  |  |
| 61. Treatment duration (days) |  |  |  |  |  |
| 62. Treatment result:  ⑴symptoms did not change ⑵ getting better ⑶ getting worse ⑷other:_______ |  |  |  |  |  |
| 63. If you had been referred, the reason was ⑴self-requested ⑵Dr.’s suggestion  ⑶other’s suggestion ⑷other:_________ |  |  |  |  |  |
| 64. Have you asked to be referred but be refused by the doctor? ⑴yes ⑵no |  |  |  |  |  |
| 65. Did you use below medicine during the visit?  1. Fluoroquinolone drugs  2. Kanamycin  3. Capreomycin  4. Amikacin |  |  |  |  |  |
| 66.Expense of examination |  |  |  |  |  |
| 67.Expense of treatment |  |  |  |  |  |
| 68.Expense of transportation and accommodation |  |  |  |  |  |
| 69.Expense of accommodation |  |  |  |  |  |
| 70. Other expenses: |  |  |  |  |  |
| 71.Medical expense covered by collective medical insurance |  |  |  |  |  |

Part VI KAP

1．Do you know the policy of transportation subsidy？ ①Yes ②No（jump to 7）

2．Do you know the eligible population of receiving subsidy?

①five-guarantee family ②Elder people without children ③certified low-income family ④unemployed population、⑤farmer ⑥domestic migrant ⑦others ⑧unknown

3．Please selected the eligible population for transportation subsidy perceived

①five-guarantee family ②Elder people without children ③certified low-income family ④unemployed population、⑤farmer ⑥domestic migrant ⑦ all TB patients ⑧unknown

4．Do you know the amount of transportation subsidy? ①Yes, CNY ②No

5．Do you receive transportation subsidy？①Yes, CNY ②No( jump to 7）

6．Do you satisfied with the transportation subsidy?

①Yes

②No, (please fill in the reason)

7．Do you think transportation subsidy could improve TB patients’ health care seeking?

①very useful ②useful ③a little useful ④no useful ⑤unkow

8．Do you think transportation subsidy could improve compliance of TB treatment？

①very useful ②useful ③a little useful ④no useful ⑤unkow

9．What kind of subsidy you want to have（可多选）

①Transprotaion ②Accommodation ③Nutrition ④Working loss ⑤Clothes ⑥Medicines ⑦others

The most important one：

How many subsidy do you want to get during treatment: CNY

**Part VII expenditure during treatment course**

|  | | Month | | | | | | | | |
| --- | --- | --- | --- | --- | --- | --- | --- | --- | --- | --- |
|  | | | 1 | 2 | 3 | 4 | 5 | 6 | 7 | 8 |
| Diagnosis and treatment | |  | |  |  |  |  |  |  |  |
| transportation | patient |  | |  |  |  |  |  |  |  |
|  | accompany | |  |  |  |  |  |  |  |  |
| accommodation | patient |  | |  |  |  |  |  |  |  |
|  | accompany | |  |  |  |  |  |  |  |  |
| Nutrition | |  | |  |  |  |  |  |  |  |
| Working loss | |  | |  |  |  |  |  |  |  |
| others | |  | |  |  |  |  |  |  |  |
| total | |  | |  |  |  |  |  |  |  |

**Annex 2: interview guideline for TB patients**

1 Basic information

Self-introduction ( including Age, education, occupation, etc)

2 Knowledge, attitude, behaviour and perception of TB related health seeking behavior (before, on and after diagnosis)

(1) What do you think about TB?

(2) Symptom before being diagnosed?

(3) Experience of health seeking before being diagnosed as TB patient. When do you go to hospital after first symptom occurred? Where? And when were you diagnosed as TB patients? How many time of hospital visits before diagnose?

(4) Experience and history of anti-TB treatment. What kind of drug did you take each time? What kind of examination did physician recommend to you? Do you know first line anti-TB medication? Do you know the second line anti-TB medication? Did you take any second line anti-TB drug? Why?

(5) Cost related with TB health care including medical cost (drug, examination) and non-medical direct cost such as transportation cost and accommodation costs.

3 Attitude and perception of transportation subsidy

(1) Economic burden of transportation

(2) Ability of payment

(3) Awareness of the “transportation subsidy initiative to poor TB patients”

(4) Perceptions in the amount of subsidy, the targeting population for subsidy and the distribution of subsidy

(5) Patients perspective of effects of transportation subsidy

(6)Satisfactions towards the “transportation subsidy initiative to poor TB patients”
